# Intra‐Oral Lasering for the Treatment of Nasolabial Folds, Marionette Lines, and Jowls: Case Examples and Protocol Methodology

**DOI:** 10.1111/jocd.70890

**Published:** 2026-05-05

**Authors:** Richard J. Miron, Elsa McGilvray, Ana De Gomez Paz, Paras Ahmad

**Affiliations:** ^1^ Department of Periodontology University of Bern Bern Switzerland; ^2^ Department of Research Advanced PRF Education Jupiter Jupiter Florida USA; ^3^ Center for Advanced Rejuvenation and Esthetics Jupiter Jupiter Florida USA; ^4^ Private Practice Lisbon Portugal

**Keywords:** CO_2_, Er‐ YAG, intraoral lasering, Nd‐ YAG, Smoothlase

## 1 Introduction

Laser therapy has become one of the fastest‐growing modalities for the management of aging skin, including the treatment of fine lines and deep wrinkles [1]. Over the years, various wavelengths have been utilized, each with distinct advantages and disadvantages, tissue penetration depths, and energy distribution characteristics. While the lower third of the face has been a problematic area for many patients with respect to facial aging, clinicians have begun to treat deep nasolabial folds, marionette lines, and jowls not only using standard extra‐oral protocols but also through innovative intra‐oral applications.

The aim of this clinical commentary was to describe for the first time the development of intra‐oral lasering modalities using Neodymium‐doped Yttrium Aluminum Garnet (Nd:YAG), Erbium‐doped Yttrium Aluminum Garnet (Er:YAG), and carbon dioxide (CO_2_) lasers. Case examples are presented utilizing novel protocols described utilizing Nd:YAG, Er:YAG, and CO_2_ lasers. Exact protocols with their respective advantages and disadvantages are documented alongside before‐and‐after outcomes.

## 2 Intra‐Oral Laser Applications—The Science Behind

### 2.1 Uses of Nd:YAG and Er:YAG Lasers

Intra‐oral lasering has emerged as a targeted protocol for the treatment of deep nasolabial folds and marionette lines. Given that the lower third of the face is often a challenging area to treat, clinicians hypothesized that combining extra‐oral and intra‐oral laser application could deliver more energy to these relatively thin tissue regions.

Interestingly, several studies have already highlighted that lasers can induce soft palate tightening to increase airway space in individuals who are prone to snoring or obstructive sleep apnea [2, 3, 4, 5]. These therapies leverage the photothermal capabilities of Neodymium‐doped Yttrium Aluminum Garnet (Nd:YAG) and Erbium‐doped Yttrium Aluminum Garnet (Er:YAG) lasers to stimulate new collagen formation in the soft palate and other mucosal tissues [2, 3, 4].

The Nd:YAG laser is a deep penetrating, non‐ablative laser capable of photobiomodulation, stimulating tissue regeneration across multiple layers [6, 7, 8, 9]. Interestingly, a non‐contact Er:YAG laser modality (Smoothmode) was proposed to deliver heat energy sufficient to tighten tissues without ablation [10]. These non‐ablative therapies have long been utilized successfully for female urinary incontinence, demonstrating their safety and efficacy on other mucosal tissues [11, 12, 13, 14]. Building on these applications, the concept of intra‐oral non‐ablative therapy was adapted for soft tissues in the lower face to enhance nasolabial folds and marionette lines [15, 16, 17, 18, 19, 20].

The Smoothmode technology consists of a series of sub‐ablative micro pulses of Er:YAG laser energy [10]. These brief temperature pulses at the mucosal surface are transformed via heat diffusion into a prolonged thermal pulse within deeper connective tissue layers [2]. This induces two complementary regenerative processes: [1] an indirect triggering effect by short‐duration heat shock of the mucosal tissue; and [2] a direct, slow thermal effect on the connective tissues [2].

Both processes stimulate collagen remodeling and neocollagenesis. Histological assessment in an animal model of the soft palate showed mucosal shrinkage without bleeding, severe inflammation, carbonization, or necrosis [21]. In addition, a pilot clinical study by Lee et al. [16] showed that the photothermal effects of intra‐oral Er:YAG lasering significantly increased the airway space/volume and minimal cross‐sectional area at 12 weeks post‐laser treatment, as measured by three‐dimensional Cone Beam Computed Tomography (CBCT).

Given their minimal invasiveness, non‐ablative intra‐oral therapies represent an ideal complement to extra‐oral laser therapy, as the two approaches do not interfere with one another. The Smoothlase protocol combines Nd:YAG and Er:YAG lasers to improve skin elasticity, tone, and texture in a minimally invasive manner. Initially, the Nd:YAG laser is applied to pre‐heat tissues to approximately 40°C in distinct patterns, followed by the Er:YAG laser delivered in a proprietary “Smoothmode” pulse sequence (Fotona). Smoothmode delivers a rapid burst of pulses over a short period, creating deep tissue heating, immediate collagen contraction, and progressive tissue tightening [22, 23].

### 2.2 Fractional CO_2_ Lasers and Their Impact on Mucosa

Fractional non‐ablative carbon dioxide (CO_2_) lasers also represent a significant advancement in mucosal tissue therapy, offering effective remodeling with minimal downtime [24, 25]. Using a fractional delivery system, these lasers create microthermal treatment zones (MTZs) [26, 27], that leave surrounding tissue intact while inducing controlled thermal injury in deeper mucosal layers. Similar to Smoothmode Er:YAG technology, their therapeutic effect relies on promoting collagen synthesis, elastin remodeling, and overall tissue regeneration through heat‐induced cellular stress [28, 29, 30]. Unlike traditional ablative CO_2_ lasers, which remove both superficial and deeper tissue layers, fractional CO_2_ lasers preserve much of the surface epithelium, allowing for faster recovery while maintaining strong clinical efficacy [31, 32].

At the cellular level, the benefits of fractional CO_2_ lasers are mediated by the activation of heat shock proteins (HSPs) and pro‐inflammatory cytokines [33, 34, 35]. Thermal exposure triggers the upregulation of HSP25, HSP47, HSP70, HSP72, and HSP90, which protect cells from further thermal damage, facilitate protein repair, and support survival [33, 34, 35]. The response enhances extracellular matrix (ECM) remodeling, specifically collagen type I and III synthesis, which are critical for structural integrity [36, 37]. Furthermore, laser‐induced thermal stress stimulates cytokines, such as interleukin‐1 (IL‐1) [38] and transforming growth factor‐beta (TGF‐β) [38], promoting angiogenesis, fibroblast activation, and collagen deposition, all of which accelerate mucosal tissue regeneration [39, 40, 41, 42].

Fractional non‐ablative and ablative CO_2_ lasers differ in depth and mechanism of action. Ablative systems vaporize epithelial and lamina propria tissues, producing deeper micro‐ablative zones, more extensive tissue removal, and pronounced resurfacing [31, 43, 44, 45, 46, 47]. Non‐ablative systems instead generate controlled thermal injury without extensive epithelial loss, sparing the tissue framework and reducing downtime and complications. Both stimulate wound healing and collagen synthesis, but the non‐ablative approach typically balances efficacy with safety, making it especially suited for mucosal applications where barrier preservation is critical.

From a wavelength perspective, the CO_2_ laser (10 600 nm) offers distinct advantages over the Er:YAG lasers (2940 nm). Both target water, the principal component of mucosa, but the CO_2_ lasers disperse heat more deeply, achieving effective coagulation and collagen stimulation in both superficial and deeper layers. In contrast, Er:YAG energy is more strongly absorbed at the surface, limiting penetration and confining its effect to remodeling [48, 49, 50]. While Er:YAG lasers excel at precise ablation and superficial resurfacing, CO_2_ lasers are preferred when deeper structural remodeling is required, such as in mucosal rejuvenation that affects nasolabial folds and marionette lines.

In summary, fractional CO_2_ lasers enhance mucosal regeneration through HSP‐mediated cytoprotection and cytokine‐regulated healing pathways. Furthermore, their deeper tissue penetration compared with Er:YAG lasers enables robust collagen and elastin remodeling across multiple tissue layers, making them a versatile and effective tool for both esthetic and functional mucosal treatments.

## 3 Case Examples and Protocols With Various Wavelengths

**FIGURE 1:**
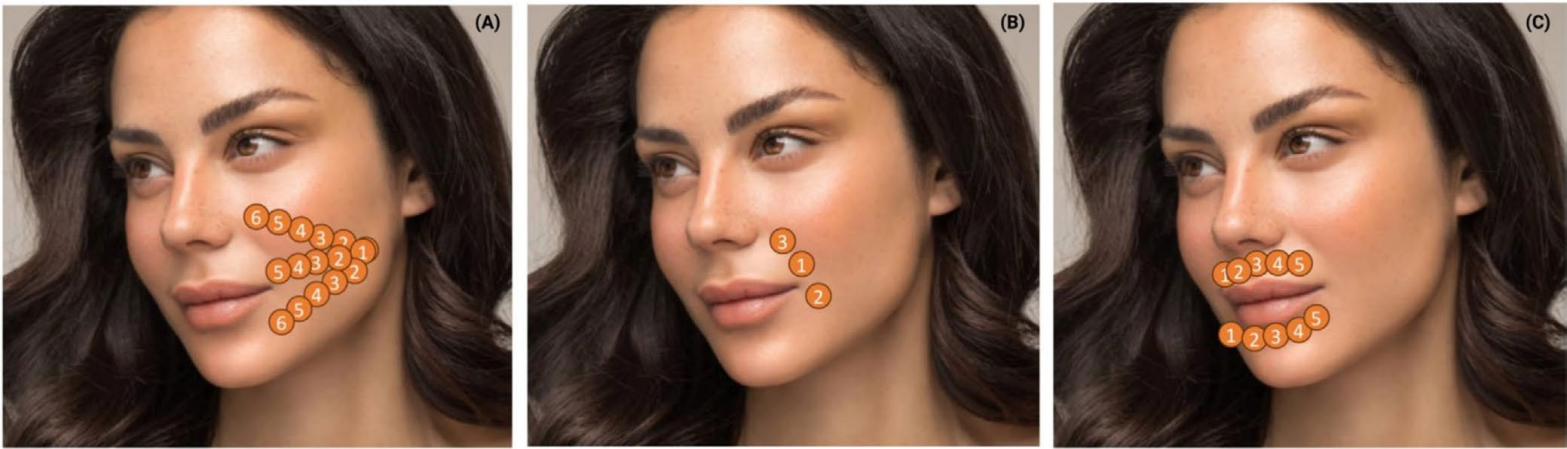
(A) Intraoral lasering pattern. When each of these protocols is applied, the clinician will begin the vector at point 1 and continue along the vector with the described protocols until reaching the final point. Generally, a 15% overlap in the laser protocols is recommended. A total of 3 vectors is generally recommended intraorally. (B) Intraoral lasering of the nasolabial fold and Marionette line. Generally, the lower portion of the nasolabial fold is treated first, followed by the marionette line and lastly the upper portion of the nasolabial fold. (C) Intraoral lasering of the perioral region to treat rhytides in the area (Smoker's lines). Both the upper and lower regions are treated separately according to the manufacturer's recommendations.

Both demo patients signed an informed written patient consent as per journal guidelines. The authors declare that the investigations were carried out following the rules of the Declaration of Helsinki of 1975, which was revised in 2013. An IRB was not needed for this study due to the retrospective nature of this study (Sterling IRB). Clinical protocols for intra‐oral laser applications—whether using Nd:YAG, Er:YAG, or CO_2_ lasers—generally follow similar treatment patterns. A typical procedure initiates with a series of passes delivered along three intraoral vectors (Figure 1A). The clinician begins treatment at the designated starting point (Point 1) and proceeds sequentially to the end point (Point 6), with adjustments made according to patient anatomy. To minimize the risk of thermal accumulation, a maximum of 15% overlap between adjacent tissue points is recommended. While three treatment vectors represent the standard approach, additional passes may be incorporated depending on the clinical severity.

Following completion of these intraoral passes, attention is directed toward the nasolabial folds and marionette lines (Figure 1B). Standard treatment typically requires two zones for the nasolabial fold and one for the marionette line, using a 7 mm spot size. Since this area is more sensitive than the intraoral mucosa, laser energy is usually reduced by around 25%. Treatment is usually sequenced beginning with the lower portion of the nasolabial fold, followed by the marionette line, and concluding with the upper portion of the fold. This sequence allows for adequate thermal relaxation of the nasolabial fold and prevents overheating, especially relevant in patients with smaller intraoral regions.

Finally, the mucosal surfaces corresponding to perioral rhytides, commonly known as smoker's lines, are addressed according to manufacturer‐specific protocols (Figure 1C). Both lower and upper regions are treated; nonetheless, direct application to the lips is avoided owing to heightened sensitivity and the potential for unintended lip plumping.

### 3.1 Treatment Protocol Using the Nd:YAG and Er:YAG Laser Combination

**FIGURE 2:**
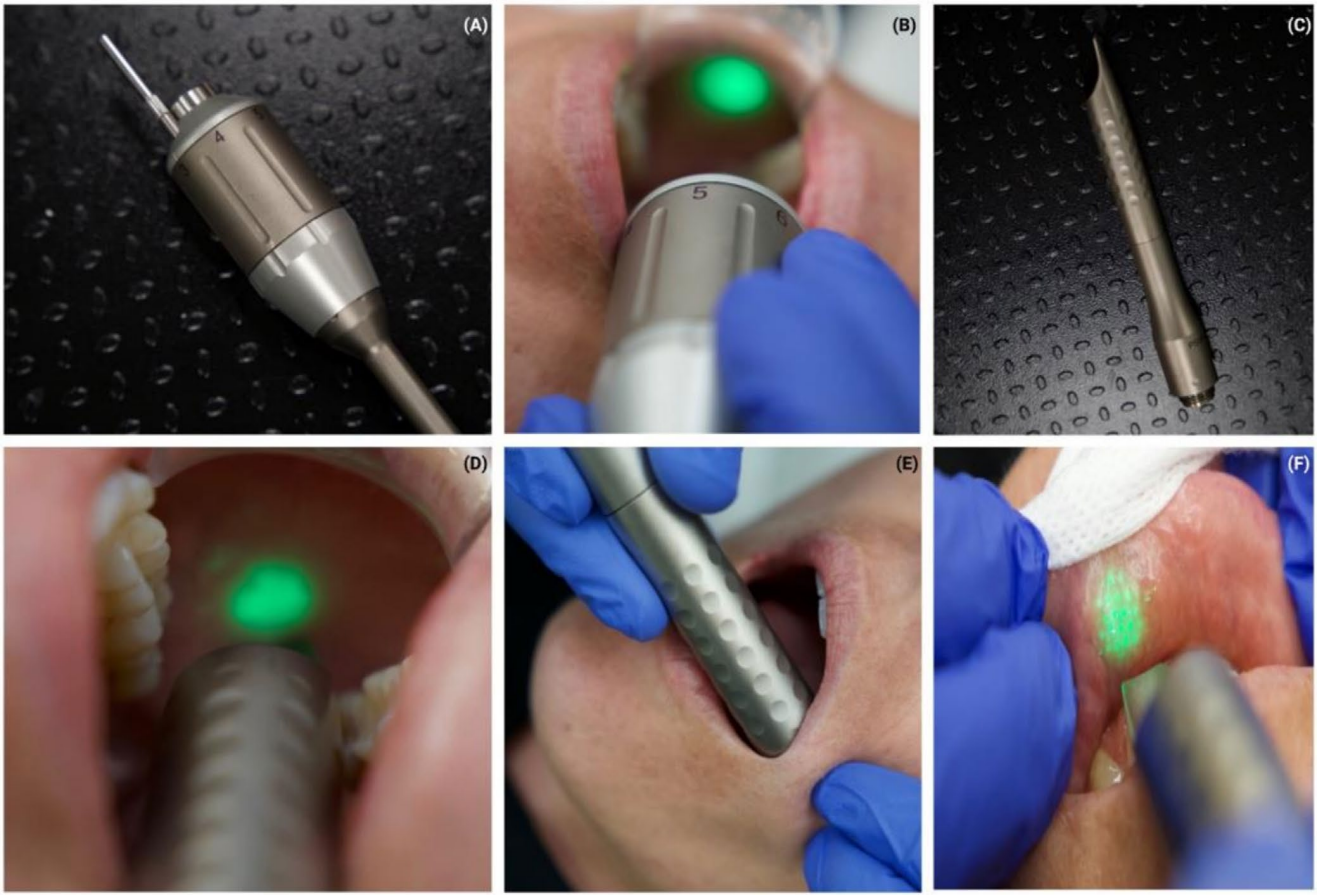
(A) An R30 handpiece is utilized to fire Nd:YAG laser (Fotona). (B) Use of the R30 handpiece to deliver Nd:YAG laser intraorally along the three vectors depicted in Figure 1. (C) PSO4 handpiece utilized to deliver Er:YAG energy (spot size = 7 mm). (D) Use of the fractional Er:YAG to deliver 6 stacks/4 passes along 3 vectors intraorally on the mucosal tissue. (E) Use of the fractional Er:YAG to deliver 6 stacks/4 passes intraorally on the nasolabial fold. (F) Use of the fractional Er:YAG to deliver 6 stacks/4 passes intraorally around the perioral region.

A novel protocol involves the sequential use of Nd:YAG and Er:YAG lasers, as illustrated in Figure 2A and demonstrated in video (QR Code 1) on a 31‐year‐old healthy ASA I female patient following informed consent. During the initial stage, the R30 handpiece is utilized with the Nd:YAG laser (1064 nm; Fotona Lightwalker, Figure 4A). Energy delivery usually consists of around 300 J/in.^2^ directed into the mucosal tissue, resulting in a total of 600 J applied per cheek. An additional 300 J may be applied intraorally around the perioral area to enhance coverage. Table 1 demonstrates the standard operating parameters, while Figure 2B illustrates the recommended handpiece positioning and aiming beam distance utilized during this protocol.

**TABLE 1:** Laser setting while utilizing Nd:YAG intra‐orally.

Parameters	Nd:YAG laser
Handpiece	R30
Spot size	2 mm
Fluence	40 J/cm^2^
Frequency	8 Hz
Energy	1200–1800 J

Following completion of Nd:YAG stage, treatment proceeds with Er:YAG laser in non‐ablative Smoothmode setting using the PS04 handpiece (7 mm spot size; collimated beam; Fotona; Figure 2C). The collimated design enables consistent spot delivery across a range of working distances, minimizing defocusing and ensuring even energy distribution.

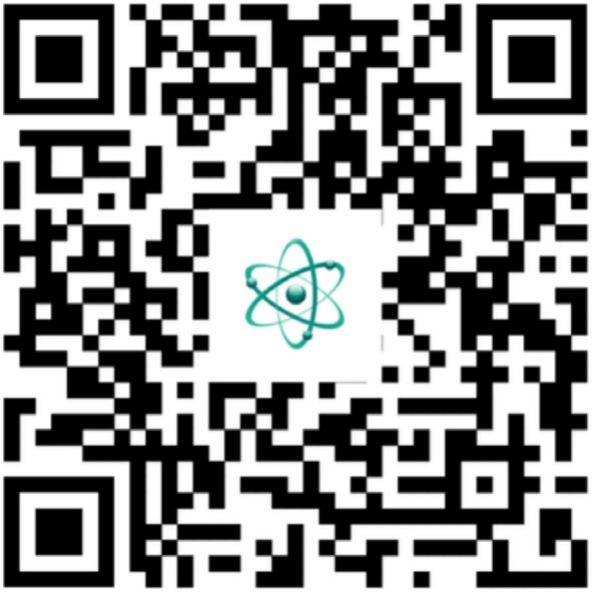

Laser parameters are set to Smoothmode with a fluence of 6–8 J/cm^2^ at 1.5–2.0 Hz, delivering six Smoothmode pulses per spot over four treatment passes (Table 2). This stage is applied systematically to three main regions: [1] the cheeks [Figure 2D] [2]; nasolabial folds and marionette lines [Figure 2E]; and [3] the perioral regions (Figure 2F). A step‐by‐step protocol is presented in QR code 1.

**TABLE 2:** Treatment of mucosal tissue using the non‐ablative Er:YAG laser on Smoothmode setting.

Parameters	Er:YAG laser
Handpiece	PSO4
Fluence	6.0–8.0 J/cm^2^
Pulse Mode	Smoothmode
Frequency	1.5–2 Hz
Stack/Spot	6 stacks/4 passes on smoothmode PULSE/SPOT
Overlap	15%
Number of pulses	2000–2300

### 3.2 Treatment Protocol Using the CO_2_ Laser

**FIGURE 3:**
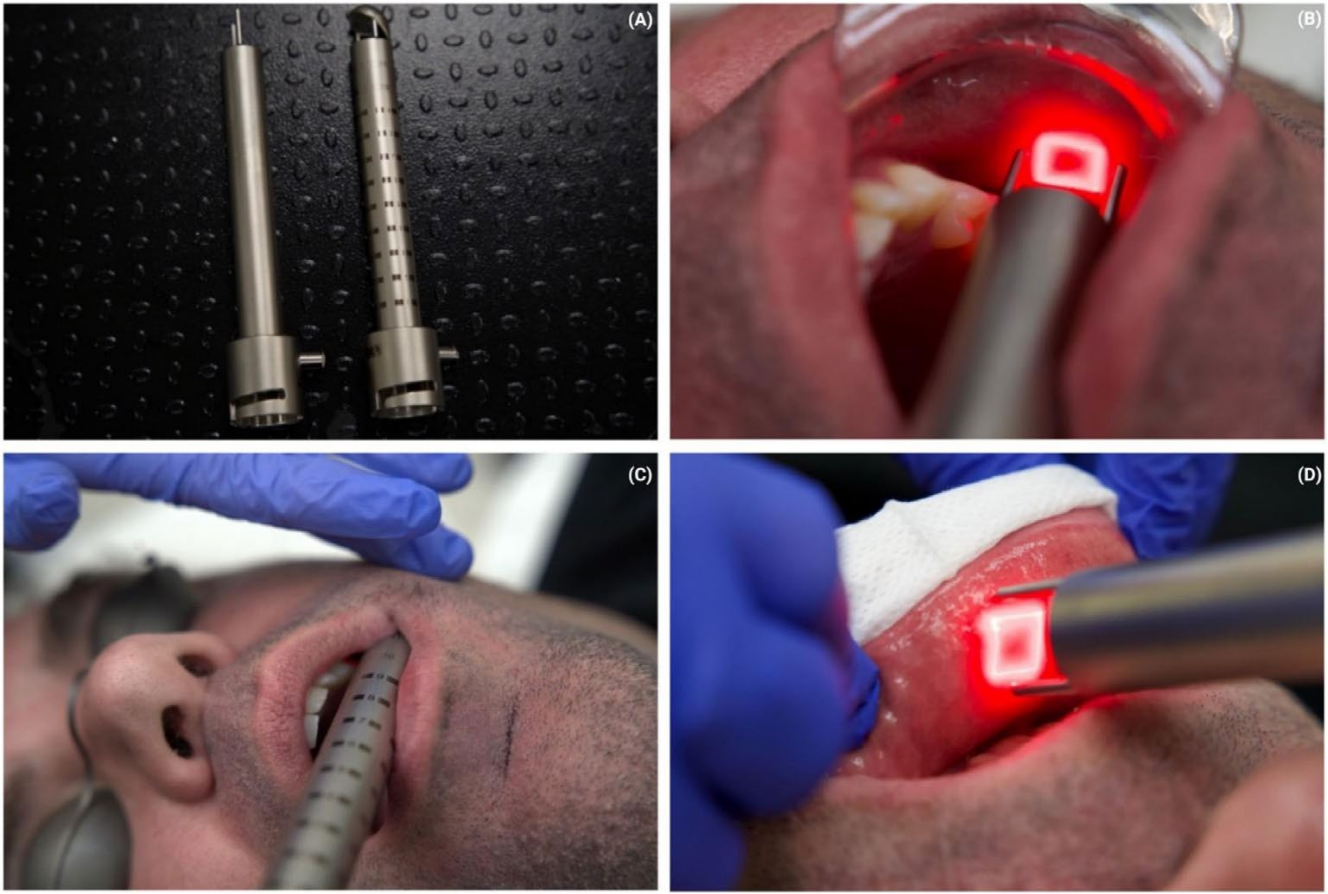
(A) Straight‐firing and side‐firing handpieces are utilized for intraoral rejuvenation with the CO_2_ laser (Deka). (B) Use of the fractional CO_2_ to deliver 2 stacks/2 passes along 3 vectors intraorally on the mucosal tissue. (C) Use of the fractional CO_2_ to deliver 2 stacks/2 passes intraorally to treat the nasolabial fold. (D) Use of the fractional CO_2_ to deliver 2 stacks/2 passes intraorally around the perioral region.

**FIGURE 4:**
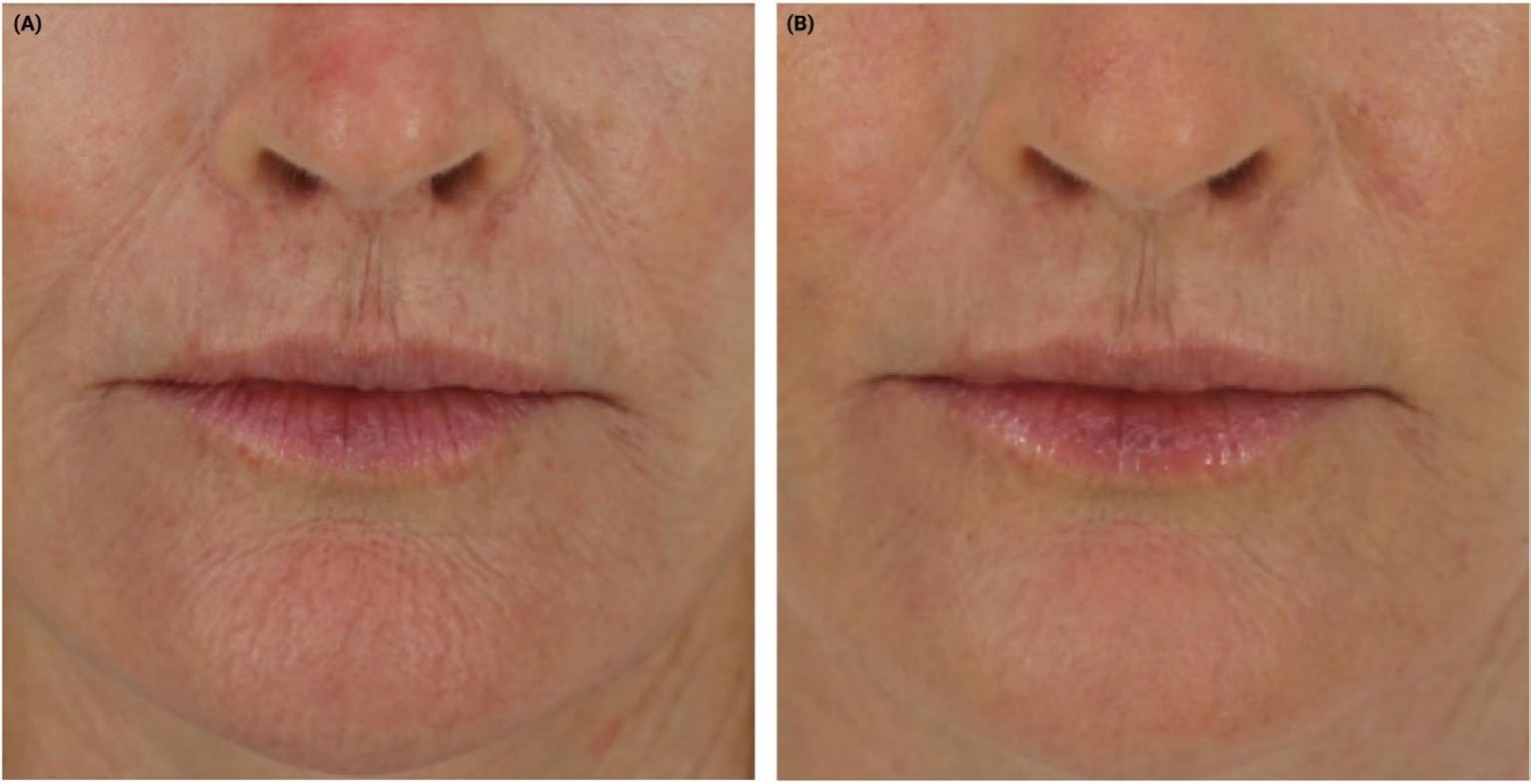
(A) Note the number of fine lines and wrinkles around the perioral region and the depth of the nasolabial folds. (B) Note the visible improvements in fine lines and wrinkles around the perioral region and the depth of the nasolabial folds following two intraoral laser therapies.

The second protocol investigated by the authors employs the CO_2_ laser, with a corresponding video presented in QR Code 2 on a healthy 41‐year‐old ASA I male patient. A straight‐firing handpiece is utilized for treatment of the cheek and perioral areas, while a side‐firing handpiece is used for the nasolabial folds and marionette lines (Deka; Figure 3A). Typical parameters include 4–6 W power with a dwell time of 1200 μs and spacing of 500 μm (Table 3). These handpieces are systematically applied to the cheeks (Figure 3B), nasolabial folds/marionette lines (Figure 3C), and perioral region (Figure 3D). A step‐by‐step protocol is presented in QR code 2. Figure 4B demonstrates a before and after clinical case incorporating these therapies.

**TABLE 3:** Treatment of mucosal tissue using the non‐ablative CO_2_ laser.

Parameters	CO_2_ laser
Handpiece	Intraoral fractional handpiece
Power	4–6 W
Dwell time	1200
Spacing	500
Scan mode	Normal/SmartTrack
Pulse mode	DP
Fluence	2.7 J/cm^2^
DOT energy	21.8 mJ
Density	15%

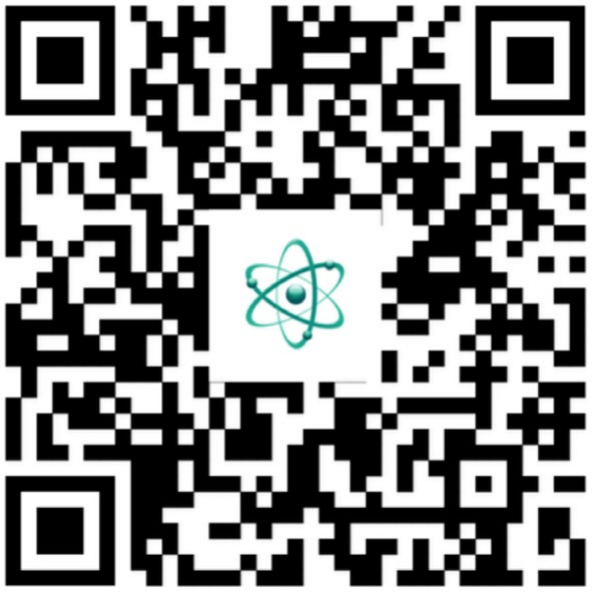

## 4 Limitations

While this article highlights recent advancements in intraoral laser therapy in facial esthetics, it is noteworthy to highlight that these case reports provide only preliminary observations and do not constitute scientific validation. There is a lack of well‐designed clinical studies investigating the use of intra‐oral lasering with no randomized clinical studies to date evaluating patient‐reported outcomes and/or clinical photos/scans. Therefore, well‐conducted randomized clinical studies are needed to evaluate the effectiveness of intraoral lasering when compared to conventional extraoral fractional lasers, fillers, or surgical lifting procedures. Notably, while both protocols available to date utilizing intra‐oral lasering focus on the non‐ablative nature of the developed protocol, no study to date has assessed the safety and efficacy with proper clinical data over a large patient population. Therefore, additional well‐designed studies with long‐term follow‐up are needed with proper documentation, validation, and standardization. Furthermore, no study to date has evaluated potential adverse events, inclusion/exclusion criteria for treatment, or potential contraindications of such intra‐oral lasering. A structured comparison of outcomes, safety profiles, and long‐term efficacy across different wavelengths would be valuable and should be studied in future RCTs. Furthermore, it would be rationale to have differences in depth of penetration of the lasers extra‐orally versus intra‐orally along with histological evaluation as to which facial layers are optimally being targeted.

## 5 Conclusion

This article highlights recent advancements in intraoral laser therapy for facial esthetics. Although further research is needed to evaluate the effectiveness of individual protocols and combination approaches, current evidence suggests that intraoral energy delivery is both safe and well‐tolerated, particularly when integrated with complementary therapies. Once regarded as an approach associated with high morbidity and prolonged downtime, laser therapy has evolved substantially, with contemporary systems providing favorable outcomes and minimal recovery periods. Looking ahead, comparative randomized clinical studies will be essential to establish optimized guidelines and standardized protocols for intraoral applications. Collectively, these advancements point toward a new era of biologically driven, minimally invasive esthetic therapies.

## Author Contributions

Conceptualization, R.J.M., E.M., A.D.G.P., P.A.; Methodology, R.J.M., E.M., and P.A.; formal analysis, R.J.M., and A.D.M.; investigation, R.J.M., E.M.; resources, R.J.M., E.M., A.D.G.P., P.A.; writing – original draft preparation, R.J.M., E.M., and P.A.; writing – review and editing, R.J.M., E.M., A.D.G.P., P.A.; supervision, A.D.G.P. and P.A.; project administration, R.J.M., E.M., A.D.G.P., P.A. All authors have read and agreed to the published version of the manuscript.

## Funding

The authors have nothing to report.

## Ethics Statement

The authors have nothing to report.

## Consent

Informed consent was provided prior to blood draw to conduct the outlined experiments. Photo patient consents were received from all patients according to Wiley standards.

## Conflicts of Interest

The authors declare no conflicts of interest.

## Data Availability

The data that support the findings of this study are available from the corresponding author upon reasonable request.

## References

[1] R. J. Miron and C. Davies , “Rejuvenating Facial Esthetics With Regenerative and Biocompatible Techniques: Part 1: Applications and Opportunities for Cosmetic Dentists. Journal of Cosmetic,” Journal of Cosmetic Dentistry 38, no. 3 (2022): 44.

[2] V. A. Picavet , M. Dellian , E. Gehrking , A. Sauter , and K. Hasselbacher , “Treatment of Snoring Using a Non‐Invasive Er: YAG Laser With SMOOTH Mode (NightLase): A Randomized Controlled Trial,” European Archives of Oto‐Rhino‐Laryngology 280, no. 1 (2023): 307–312. 35867153 10.1007/s00405-022-07539-9 PMC9813098

[3] T. Unver , A. Usumez , E. Aytugar , T. Kıran , and A. Üşümez , “Histological Effects of NightLase in the Soft Palate of Rats: A Pilot Study,” J Laser Health Acad 2015 (2015): 1–3.

[4] D. Chua , S. B. Nicholas , T. S. Lim , et al., “Noninvasive Er: YAG Laser With SMOOTH Mode (Nightlase) for Snoring,” Journal of Craniofacial Surgery Open 3, no. 2 (2025): e0030.

[5] A. N. Kassab , A. El Kharbotly , A. Abd Elsamie , and M. R. Ahmed , “A Long‐Term Follow‐Up Study for the Treatment of Snoring After Using Patterned Non‐Ablative Erbium: YAG 2,940 Nm Laser,” International Archives of Otorhinolaryngology 27, no. 1 (2023): e104–e110. 36714903 10.1055/s-0042-1744171 PMC9879649

[6] N. L. T. Hang , A. E. Aviña , C.‐J. Chang , and T.‐S. Yang , “Photobiomodulation in Promoting Cartilage Regeneration,” International Journal of Molecular Sciences 26, no. 12 (2025): 5580. 40565045 10.3390/ijms26125580 PMC12193606

[7] M. Gryka‐Deszczyńska , Z. Grzech‐Leśniak , D. Dembicka‐Mączka , et al., “Effects of Er: YAG and Nd: YAG Lasers With Photobiomodulation on Alveolar Bone Preservation Post‐Extraction: A Randomized Clinical Control Trial,” Photonics 12, no. 8 (2025): 817.

[8] S.‐Y. Chang , N. T. Carpena , B. J. Kang , and M. Y. Lee , “Effects of Photobiomodulation on Stem Cells Important for Regenerative Medicine,” Medical Lasers; Engineering, Basic Research, and Clinical Application 9, no. 2 (2020): 134–141.

[9] E. J. Khamees , N. N. Jawad , and H. M. Azeez , “The Use of Lasers (Ablative Laser, Non‐Ablative Laser, Fractional Laser, Photobiomodulation (PBM)) in Skin Regeneration,” International Journal of Biological, Physical and Chemical Dent Stud 4, no. 1 (2022): 7–13.

[10] M. Lukac , A. Zorman , N. Lukac , T. Perhavec , and B. Tasic , “Characteristics of Non‐Ablative Resurfacing of Soft Tissues by Repetitive Er: YAG Laser Pulse Irradiation,” Lasers in Surgery and Medicine 53, no. 9 (2021): 1266–1278. 33792949 10.1002/lsm.23402 PMC8518959

[11] N. Fistonić , I. Fistonić , Š. F. Guštek , et al., “Minimally Invasive, Non‐Ablative Er: YAG Laser Treatment of Stress Urinary Incontinence in Women—A Pilot Study,” Lasers in Medical Science 31, no. 4 (2016): 635–643. 26861984 10.1007/s10103-016-1884-0 PMC4851697

[12] J. I. Pardo , V. R. Sola , and A. A. Morales , “Treatment of Female Stress Urinary Incontinence With Erbium‐YAG Laser in Non‐Ablative Mode,” European Journal of Obstetrics & Gynecology and Reproductive Biology 204 (2016): 1–4. 27448169 10.1016/j.ejogrb.2016.06.031

[13] L. Hympanova , K. Mackova , M. El‐Domyati , et al., “Effects of Non‐Ablative Er: YAG Laser on the Skin and the Vaginal Wall: Systematic Review of the Clinical and Experimental Literature,” International Urogynecology Journal 31, no. 12 (2020): 2473–2484. 32780174 10.1007/s00192-020-04452-9

[14] M. Blaganje , D. Šćepanović , L. Žgur , I. Verdenik , F. Pajk , and A. Lukanović , “Non‐Ablative Er: YAG Laser Therapy Effect on Stress Urinary Incontinence Related to Quality of Life and Sexual Function: A Randomized Controlled Trial,” European Journal of Obstetrics & Gynecology and Reproductive Biology 224 (2018): 153–158. 29604548 10.1016/j.ejogrb.2018.03.038

[15] E. A. Cetinkaya , M. Turker , K. Kiraz , and H. K. Gulkesen , “Er: Yag Laser Treatment of Simple Snorers in an Outpatient Setting,” Orl 78, no. 2 (2016): 70–76. 26967167 10.1159/000443510

[16] C. Y. Lee and C. C. Lee , “Evaluation of a Non‐Ablative Er: YAG Laser Procedure to Increase the Oropharyngeal Airway Volume: A Pilot Study,” Dent Oral Craniofac Res 1, no. 3 (2015): 56–59.

[17] I. F. Storchi , S. Parker , F. Bovis , S. Benedicenti , and A. Amaroli , “Outpatient Erbium: YAG (2940 Nm) Laser Treatment for Snoring: A Prospective Study on 40 Patients,” Lasers in Medical Science 33, no. 2 (2018): 399–406. 29333582 10.1007/s10103-018-2436-6

[18] H. Frelich , W. Ścierski , M. Marków , J. Frelich , H. Frelich , and M. Maciej , “Minimally Invasive Erbium Laser Treatment for Selected Snorers,” Lasers in Medical Science 34, no. 7 (2019): 1413–1420. 30762193 10.1007/s10103-019-02731-6

[19] L. Monteiro , A. Macedo , L. Corte‐Real , F. Salazar , and J.‐J. Pacheco , “Treatment of Snoring Disorder With a Non‐Ablactive Er: YAG Laser Dual Mode Protocol. An Interventional Study,” Journal of Clinical and Experimental Dentistry 12, no. 6 (2020): e561. 32665815 10.4317/jced.56953 PMC7335611

[20] C. Neruntarat , K. Khuancharee , and P. Shoowit , “Er: YAG Laser for Snoring: A Systemic Review and Meta‐Analysis,” Lasers in Medical Science 35, no. 6 (2020): 1231–1238. 32112250 10.1007/s10103-020-02987-3

[21] T. Unver , E. Aytugar , O. Ozturan , T. Kıran , E. Ademci , and A. Usumez , “Histological Effects of Er: YAG Laser Irradiation With Snoring Handpiece in the Rat Soft Palate,” Photomedicine and Laser Surgery 34, no. 8 (2016): 321–325. 27196421 10.1089/pho.2015.4044

[22] H. M. Ebrahim and K. Gharib , “Correction of Nasolabial Folds Wrinkle Using Intraoral Non‐Ablative Er: YAG Laser,” Journal of Cosmetic and Laser Therapy 20, no. 6 (2018): 364–368. 29482388 10.1080/14764172.2018.1439964

[23] A. Gaspar , “Fotona VectorLift Technique for Eyebrow Tail Elevation and Upper Eyelid Rejuvenation With Hyperstacking of Smooth Mode Pulses,”.

[24] J. Liboon , W. Funkhouser , and D. J. Terris , “A Comparison of Mucosal Incisions Made by Scalpel, CO2 Laser, Electrocautery, and Constant‐Voltage Electrocautery,” Otolaryngology–Head and Neck Surgery 116, no. 3 (1997): 379–385. 9121794 10.1016/S0194-59989770277-8

[25] Y. Daigo , E. Daigo , H. Fukuoka , et al., “CO2 Laser for Esthetic Healing of Injuries and Surgical Wounds With Small Parenchymal Defects in Oral Soft Tissues,” Diseases 11, no. 4 (2023): 172. 38131978 10.3390/diseases11040172 PMC10742548

[26] M. F. Marqa and S. Mordon , “Laser Fractional Photothermolysis of the Skin: Numerical Simulation of Microthermal Zones,” Journal of Cosmetic and Laser Therapy 16, no. 2 (2014): 57–65. 24410612 10.3109/14764172.2013.854642

[27] F. E. Stephan , M. B. Habre , J. F. Helou , R. G. Tohme , and R. R. Tomb , “Fractional CO2 Laser Treatment for a Skin Graft,” Journal of Cosmetic and Laser Therapy 18, no. 1 (2016): 46–47. 26052811 10.3109/14764172.2015.1052508

[28] R. E. Fitzpatrick , E. F. Rostan , and N. Marchell , “Collagen Tightening Induced by Carbon Dioxide Laser Versus Erbium: YAG Laser,” Lasers in Surgery and Medicine: The Official Journal of the American Society for Laser Medicine and Surgery 27, no. 5 (2000): 395–403. 10.1002/1096-9101(2000)27:5<395::AID-LSM1000>3.0.CO;2-4 11126433

[29] R. S. C. Bueno , G. L. Carvalho , É. V. G. Puccia , et al., “Photobiomodulation as a Modulator of Collagen Remodeling Following Fractional CO_2_ Laser Therapy,” Lasers in Medical Science 40, no. 1 (2025): 1–8. 10.1007/s10103-025-04581-x 40789786

[30] J. S. Orringer , S. Kang , T. M. Johnson , et al., “Connective Tissue Remodeling Induced by Carbon Dioxide Laser Resurfacing of Photodamaged Human Skin,” Archives of Dermatology 140, no. 11 (2004): 1326–1332. 15545540 10.1001/archderm.140.11.1326

[31] T. Omi and K. Numano , “The Role of the CO2 Laser and Fractional CO2 Laser in Dermatology,” Laser Therapy 23, no. 1 (2014): 49–60. 24771971 10.5978/islsm.14-RE-01 PMC3999431

[32] P. J. Carniol , S. Harirchian , and E. Kelly , “Fractional CO2 Laser Resurfacing,” Facial Plastic Surgery Clinics 19, no. 2 (2011): 247–251. 21763986 10.1016/j.fsc.2011.05.004

[33] C. Hu , J. Yang , Z. Qi , et al., “Heat Shock Proteins: Biological Functions, Pathological Roles, and Therapeutic Opportunities,” MedComm 3, no. 3 (2022): e161. 35928554 10.1002/mco2.161 PMC9345296

[34] A. Yamasaki , H. Ito , J. Yusa , Y. Sakurai , N. Okuyama , and R. Ozawa , “Expression of Heat Shock Proteins, Hsp70 and Hsp25, in the Rat Gingiva After Irradiation With a CO2 Laser in Coagulation Mode,” Journal of Periodontal Research 45, no. 3 (2010): 323–330. 19909401 10.1111/j.1600-0765.2009.01239.x

[35] R. Ferrando , S. Schuschereba , J. Quong , and P. Bowman , “Carbon Dioxide Laser Induction of Heat Shock Protein 70 Synthesis: Comparison With High Temperature Treatment,” Lasers in Medical Science 10, no. 3 (1995): 207–212.

[36] M. González‐Ramos , L. Calleros , S. López‐Ongil , et al., “HSP70 Increases Extracellular Matrix Production by Human Vascular Smooth Muscle Through TGF‐β1 Up‐Regulation,” International Journal of Biochemistry & Cell Biology 45, no. 2 (2013): 232–242. 23084979 10.1016/j.biocel.2012.10.001

[37] R. R. Anderson and J. A. Parrish , “Selective Photothermolysis: Precise Microsurgery by Selective Absorption of Pulsed Radiation,” Science 220, no. 4596 (1983): 524–527. 6836297 10.1126/science.6836297

[38] F. Prignano , P. Campolmi , P. Bonan , et al., “Fractional CO2 Laser: A Novel Therapeutic Device Upon Photobiomodulation of Tissue Remodeling and Cytokine Pathway of Tissue Repair,” Dermatologic Therapy 22 (2009): S8–S15. 19891690 10.1111/j.1529-8019.2009.01265.x

[39] J. Varga , J. Rosenbloom , and S. A. Jimenez , “Transforming Growth Factor β (TGF β) Causes a Persistent Increase in Steady‐State Amounts of Type I and Type III Collagen and Fibronectin mRNAs in Normal Human Dermal Fibroblasts,” Biochemical Journal 247, no. 3 (1987): 597–604. 3501287 10.1042/bj2470597 PMC1148454

[40] R. Montesano and L. Orci , “Transforming Growth Factor Beta Stimulates Collagen‐Matrix Contraction by Fibroblasts: Implications for Wound Healing,” National Academy of Sciences of the United States of America 85, no. 13 (1988): 4894–4897. 10.1073/pnas.85.13.4894 PMC280543 3164478

[41] M. S. Pepper , “Transforming Growth Factor‐Beta: Vasculogenesis, Angiogenesis, and Vessel Wall Integrity,” Cytokine & Growth Factor Reviews 8, no. 1 (1997): 21–43. 9174661 10.1016/s1359-6101(96)00048-2

[42] A. Viloria‐Petit , A. Richard , S. Zours , M. Jarad , and B. L. Coomber , “Role of Transforming Growth Factor Beta in Angiogenesis,” in Biochemical Basis and Therapeutic Implications of Angiogenesis (Springer, 2013), 23–45.

[43] X. Yue and H. Wang , “Application of Reflectance Confocal Microscopy to Investigate the Non‐Ablative, Micro‐Ablative, and Ablative Effects of CO2 Fractional Laser Irradiation on Skin,” Lasers in Medical Science 35, no. 4 (2020): 957–964. 31845041 10.1007/s10103-019-02910-5

[44] L. A. Brightman , J. A. Brauer , R. Anolik , et al., “Ablative and Fractional Ablative Lasers,” Dermatologic Clinics 27, no. 4 (2009): 479–489. 19850197 10.1016/j.det.2009.08.009

[45] J. K. Duplechain , “Fractional CO2 Resurfacing: Has It Replaced Ablative Resurfacing Techniques?,” Facial Plastic Surgery Clinics of North America 21, no. 2 (2013): 213–227. 23731583 10.1016/j.fsc.2013.02.006

[46] A. E. Ortiz , M. P. Goldman , and R. E. Fitzpatrick , “Ablative CO2 Lasers for Skin Tightening: Traditional Versus Fractional,” Dermatologic Surgery 40 (2014): S147–S151. 25417566 10.1097/DSS.0000000000000230

[47] S. Salvatore , U. Leone Roberti Maggiore , S. Athanasiou , et al., “Histological Study on the Effects of Microablative Fractional CO2 Laser on Atrophic Vaginal Tissue: An Ex Vivo Study,” Menopause 22, no. 8 (2015): 845–849. 25608269 10.1097/GME.0000000000000401

[48] R. J. Caniglia , “Erbium: YAG Laser Skin Resurfacing,” Facial Plastic Surgery Clinics of North America 12, no. 3 (2004): 373–377. 15261174 10.1016/j.fsc.2004.03.005

[49] C. Weinstein , “New Lasers for Skin Resurfacing—Erbium: YAG/CO2 Systems,” Seminars in Plastic Surgery 13, no. 3 (1999): 57–82.

[50] K. A. Khatri , V. Ross , J. M. Grevelink , C. M. Magro , and R. R. Anderson , “Comparison of Erbium: YAG and Carbon Dioxide Lasers in Resurfacing of Facial Rhytides,” Archives of Dermatology 135, no. 4 (1999): 391–397. 10206045 10.1001/archderm.135.4.391

